# Deep sleep maintains learning efficiency of the human brain

**DOI:** 10.1038/ncomms15405

**Published:** 2017-05-22

**Authors:** Sara Fattinger, Toon T. de Beukelaar, Kathy L. Ruddy, Carina Volk, Natalie C. Heyse, Joshua A. Herbst, Richard H. R. Hahnloser, Nicole Wenderoth, Reto Huber

**Affiliations:** 1 Child Development Center, University Children’s Hospital Zurich, Zurich 8032, Switzerland; 2 Neuroscience Center Zurich (ZNZ), University of Zurich, Zurich 8057, Switzerland; 3 Movement Control and Neuroplasticity Research Group, Department of Kinesiology, KU Leuven, 3001 Heverlee, Belgium; 4 Neural Control of Movement Lab, Department of Health Sciences and Technology, ETH Zurich, Zurich 8057, Switzerland; 5 Institute of Neuroinformatics, University of Zurich and ETH Zurich, Zurich 8057, Switzerland; 6 Department of Child and Adolescent Psychiatry and Psychotherapy, Psychiatric Hospital, University of Zurich, Zurich, Switzerland

## Abstract

It is hypothesized that deep sleep is essential for restoring the brain’s capacity to learn efficiently, especially in regions heavily activated during the day. However, causal evidence in humans has been lacking due to the inability to sleep deprive one target area while keeping the natural sleep pattern intact. Here we introduce a novel approach to focally perturb deep sleep in motor cortex, and investigate the consequences on behavioural and neurophysiological markers of neuroplasticity arising from dedicated motor practice. We show that the capacity to undergo neuroplastic changes is reduced by wakefulness but restored during unperturbed sleep. This restorative process is markedly attenuated when slow waves are selectively perturbed in motor cortex, demonstrating that deep sleep is a requirement for maintaining sustainable learning efficiency.

## Abstract (web summary)

Deep sleep is hypothesized to restore the brain's capacity to learn. Here the authors provide causal evidence by specifically perturbing slow wave activity over the motor cortex during NREM sleep in humans and demonstrate a reduction in neurophysiological markers of plasticity and capacity for motor learning.

Many of us know from personal experience that a single night of low quality sleep can make mental tasks effortful and inefficient. Accordingly, one theory proposes that sleep is crucial for restoring the brain’s metabolic^1^ and neural homeostasis^2^,^3^, thus ensuring efficient functioning during the next bout of wakefulness. Environmental inputs are constantly experienced when awake and lead to a progressive increase of synaptic strength^4^,^5^,^6^. Perpetual increases in synaptic strength, however, would render the brain highly insensitive to new inputs because neurons would lose their ability to fire selectively and synapses could not be further potentiated, thus saturating neural plasticity^7^. Additionally, the need for cellular maintenance^2^ and the removal of potentially neurotoxic waste^1^ would be markedly enhanced causing an unsustainable level of energy consumption. Deep sleep in particular is thought to be essential for down-regulating synaptic strength^3^. During deep non-rapid eye movement (NREM) sleep neurons start to oscillate between a depolarized on-state when they fire and a hyperpolarized off-state when they are silent^8^. Because neurons are highly interconnected, synchronization of on- and off-states within larger neuronal assemblies drives 0.5–4.5 Hz oscillations, termed ‘slow waves’, which are typically detected in the surface electroencephalogram (EEG) or local field potentials during NREM sleep^9^. Slow wave activity (SWA, EEG power between 1 and 4.5 Hz) is highest shortly after falling asleep, that is, when the sleep need is still high, while it is markedly reduced at the end of the night after restorative processes have taken place^10^. However, until now the proposal that SWA is necessary for restorative processes has been mainly supported by correlative evidence demonstrating that neural plasticity induced while awake (for example, by practicing a specific task) leads to more SWA during sleep^11^,^12^. This effect is highly localized with SWA being significantly higher in brain areas that were activated during the task than in non-task areas, suggesting that the brain responds to a locally increased need for sleep^3^,^5^,^11^,^13^. However, in order to demonstrate that slow waves are directly responsible for restorative processes, one has to establish a causal relationship between these phenomena. Such an endeavor has thus far proved impossible due to the inability to manipulate SWA in humans on a local level. Here, we introduce a novel perturbation approach where real time closed-loop acoustic stimulation was timed to coincide precisely with the vulnerable down-phase of EEG slow waves^2^. We targeted SWA in primary motor cortex (M1), taking advantage of well-established behavioural and neurophysiological markers of motor training-induced neuroplasticity to estimate the effect of locally perturbed deep sleep in M1. We demonstrate that the capacity to undergo neuroplastic changes in response to learning is reduced by wakefulness but restored during unperturbed sleep. This restorative process is markedly attenuated when slow waves are selectively perturbed in motor cortex.

## Results

### Local slow wave perturbation

**Figure 1:**
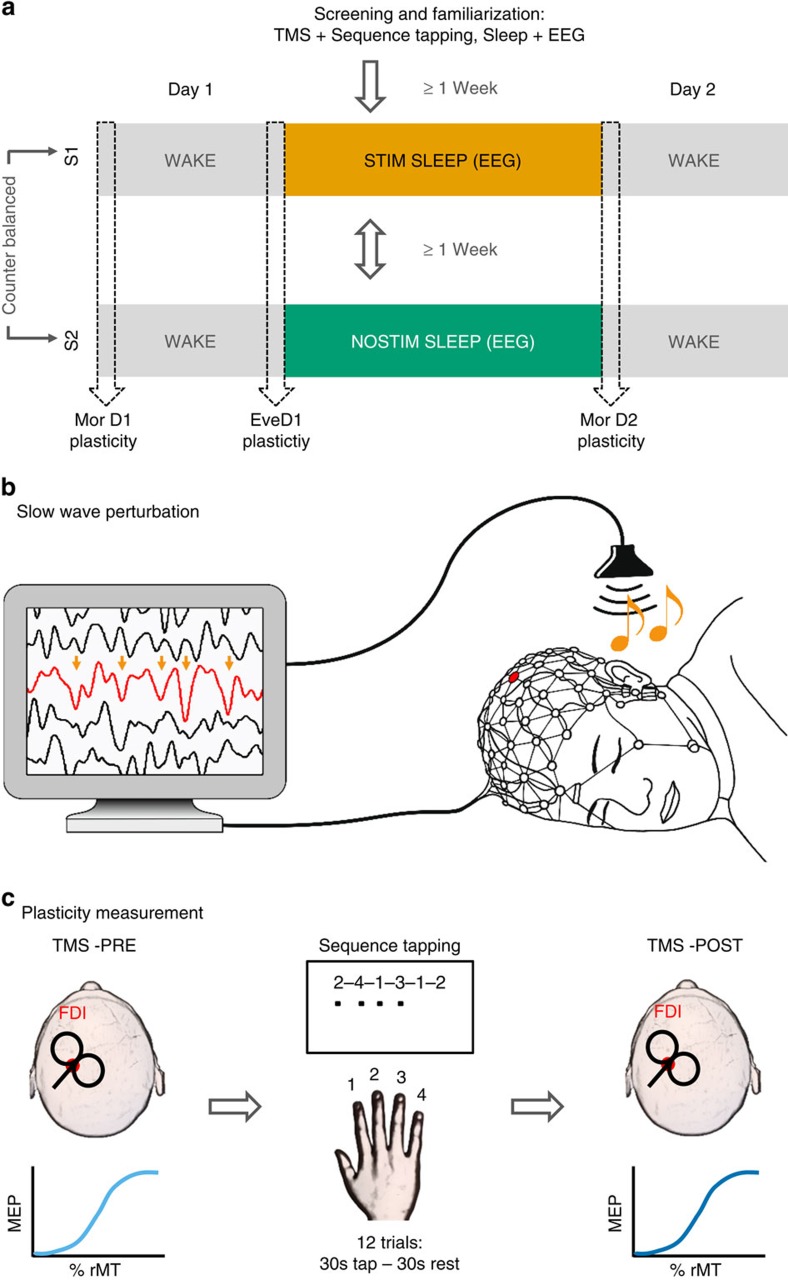
Overview of the experimental protocol. (**a**) After a familiarization session, volunteers participated in two experimental sessions. These were separated by 1 week, with both sessions including three separate learning assessments, the first in the morning of day 1 (Mor D1), the second in the evening on the same day (Eve D1), and a third in the morning the next day (Mor D2). During the night, sleep was recorded using high density EEG. In a cross-over counter balanced design, in one experimental session, slow waves were localy perturbed over left primary motor cortex by acoustic stimulation delivered time-locked to the donw-phase of sleep slow waves (STIM, yellow) while in the other experimental session no stimulation was applied (NOSTIM, green, **b**). During each learning assessment participatns were subjected to a new motor sequence training (finger taping task) and corticomotor excitability was measured via an IO curve before and after performing the motor sequence training, in order to determine changes indicative of motor plasticity as a consequence of training (**c**). (**b**) In the STIM sessions slow waves were perturbed using acoustic stimulation precisely time-locked to the down-phase of sleep slow waves (yellow arrows) detected in the electrode located closest to the sensorimotor representation of the trained hand (red electrode and signal). (**c**) During each learning assessment subjects acquired a new six-element motor sequence during 12 training trials (that is, 30 s tapping followed by 30 s rest) lasting 12 min in total. Changes in corticomotor excitability of the right first dorsal interosseus (FDI, red dot) were measured before motor training (TMS-PRE) and after (TMS-POST) via an input-output curve (IO curve).

**Figure 2:**
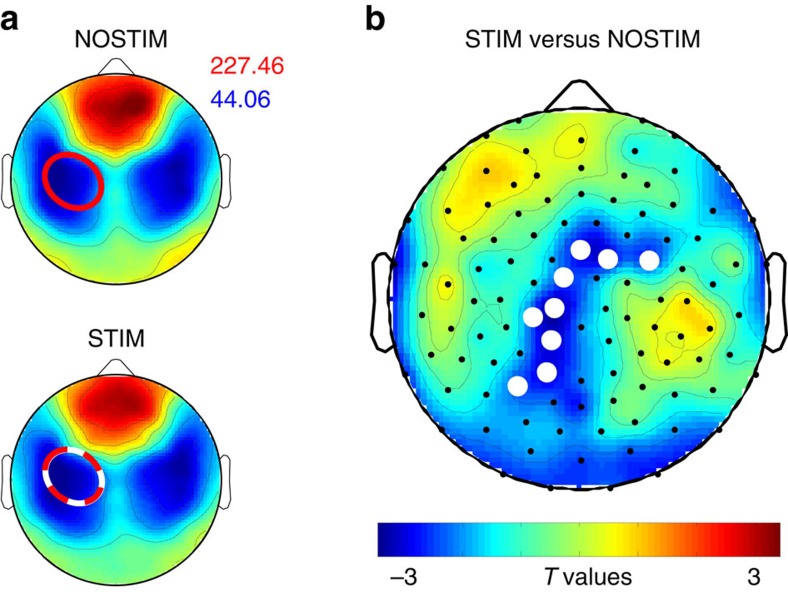
Topographical distribution of low-slow wave activity. Comparisons of low slow wave activity (low-SWA, 1–2 Hz) between NOSTIM and STIM sessions. (**a**) Topographical map of low-SWA of the two experimental nights scaled to maximum (red) and minimum (blue) power values (μV^2^/Hz). Red circles indicate the position of the TMS hotspot, the white circle indicates the position of the selected electrode for slow wave detection. Note, in the main experiment the TMS hotspot-electrodes and the selected electrodes for slow wave detection were the same. (**b**) Statistical comparison (*t*-values) of low-SWA between the STIM and NOSTIM sessions (paired *t*-test; *n*=13). Blue colours indicate a decrease and red colours an increase in low-SWA in the STIM compared to NOSTIM session. During STIM session sleep a reduction of low-SWA of 12.00±3.92% (*P*=0.009) in a local cluster of 9 electrodes over left sensory-motor area (white dots, *P*<0.05, after nonparametric cluster-based statistical testing) was found. See Methods section for further details.

**Figure 3:**
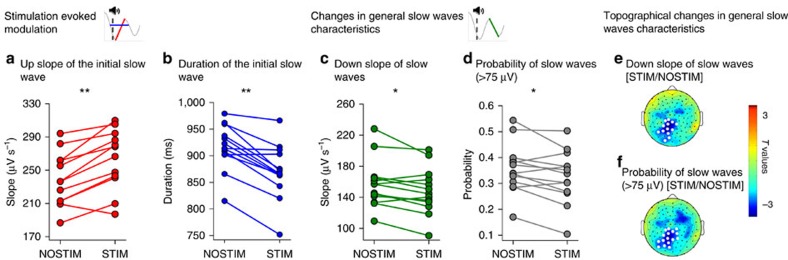
Stimulation evoked effects on slow waves. Stimulation evoked modulation and induced changes on general slow wave characteristics in the hotspot-electrode. (**a**,**b**) Stimulation evoked modulation recorded at the hotspot-electrode during the NOSTIM and STIM sessions. The immediate up-slope (red) and duration (blue) of the initial single slow wave (see schema) are shown (mean values of the NOSTIM and STIM session for each subject). (**c**,**d**) Induced changes in slow wave characteristics during sleep. The down-slope of slow waves (green, mean down-slope) and the probability of slow waves (gray, defined as all waves with a peak-to-peak amplitude larger than 75 μV) are shown (mean values of the NOSTIM and STIM session of each subject). *P<0.05, **P<0.001, paired *t*-test; *n*=13. See Methods section for further details. (**e**,**f**) Topographical distribution of the comparison (*t*-values) for changes in general slow wave characteristics (similar to **c**,**d**). (**e**) Down-slope of slow waves (mean down slope) between STIM and NOSTIM sessions. (**f**) Probability of slow waves (defined as all waves with a peak-to-peak amplitude larger than 75 μV) between STIM and NOSTIM night. Blue colours indicate a decrease and red colours an increase in the STIM compared to NOSTIM session (paired *t*-test; *n*=13, white dots, *P*<0.05, after nonparametric cluster-based statistical testing).

We report data from 13 volunteers (21.2±0.4 years of age, all right handed, 6 females) who were first familiarized with the procedures and participated then in two experimental sessions (see Supplementary Fig. 1 for subject drop-out and outlier detection). Each session consisted of three learning assessments: a new motor sequence (finger tapping) was learned on the morning of Day 1 (Mor D1), another new motor sequence was learned on the evening of Day 1 (Eve D1) which was followed by one night of sleep in the laboratory during which high-density (HD) EEG was measured, and another new motor sequence was learned on the morning of Day 2 (Mor D2, for an overview of the study design see Fig. 1). During one experimental session sleep was perturbed by acoustic stimulation triggered by the down-phase of local slow waves in M1 (STIM), while in the other session subjects were allowed a normal night of sleep (NOSTIM, Fig. 1a,b; see Methods section). Subjects were blind to this experimental manipulation and subjective sleep ratings were not significantly different between sessions (Supplementary Table 1, nor were subjective ratings of different psychological parameters (for example, focus, motivation or tiredness; Supplementary Table 2) or objective measures of vigilance (Supplementary Table 3). Acoustic stimulation was switched on during stable NREM sleep and it was triggered by slow waves in the 0.5–2 Hz frequency range. Stimulation caused a local reduction of SWA, most prominently in the low frequency range (low SWA, 1–2 Hz; Supplementary Fig. 2). We found a 12.00±3.92% power reduction in low-SWA during the STIM session compared to the NOSTIM session in a cluster of 9 electrodes (Fig. 2, for a comprehensive overview of the entire frequency range (0.5–25 Hz) Supplementary Fig. 3). Additional analyses focused on the hotspot-electrode in M1 (for details see Methods section, Fig. 1b). Low-SWA measured by this electrode was reduced by 13.4±4.12% (*P*=0.007, paired *t*-test; *n*=13). During STIM sleep we applied acoustic stimulation on 53.1±3.71% of all slow waves present during the night. This caused a significant acute effect (evoked manipulation) by steepening the slope of the up-phase and shortening the duration of the perturbed slow wave (Fig. 3a,b, *P*<0.001, paired *t*-test; *n*=13). The stimulation also changed general slow-wave characteristics throughout sleep (Fig. 3c,d). Thus, the level of synchronization necessary for the generation of large amplitude waves was reduced, and as a consequence, SWA was also reduced. This finding is also supported by the positive correlation between the slope reduction of the down-phase and the reduction in low-SWA (Spearman’s rho=0.69, *P*=0.01; *n*=13). Exploratory analyses including all channels revealed that the general effects of acoustic stimulation are locally restricted (Fig. 3e,f).

General sleep architecture was comparable between sessions (Supplementary Table 4), except for a tendency towards reduced N3 sleep during STIM sleep compared to NOSTIM sleep (NOSTIM: 130.5±10.6 min; STIM: 117.9±9.8 min; *P*=0.05; *n*=13). Global SWA (mean over all electrodes) did not differ between the two conditions (NOSTIM: 120.1±16.6 μV^2^/Hz; STIM: 114.4±16.3 μV^2^/Hz; *P*=0.16; *n*=13). These results confirm that acoustic stimulation selectively reduced low-SWA within sensorimotor areas of the trained hemisphere while the general sleep architecture was largely unaffected.

### Effects of local slow wave perturbation on neuroplasticity

Next we asked whether perturbed SWA disturbs sleep-dependent processes influencing motor learning the next day. During each experimental session neural plasticity was assessed in response to learning a new motor sequence on Mor D1, Eve D1, and Mor D2 (Fig. 1a,c). Motor learning was quantified by a Performance Score, which takes into account the speed-accuracy tradeoff (% correct sequences divided by inter-tap interval in s), and Tapping Variability (average s.d. of inter-tap intervals in completed sequences).

**Figure 4:**
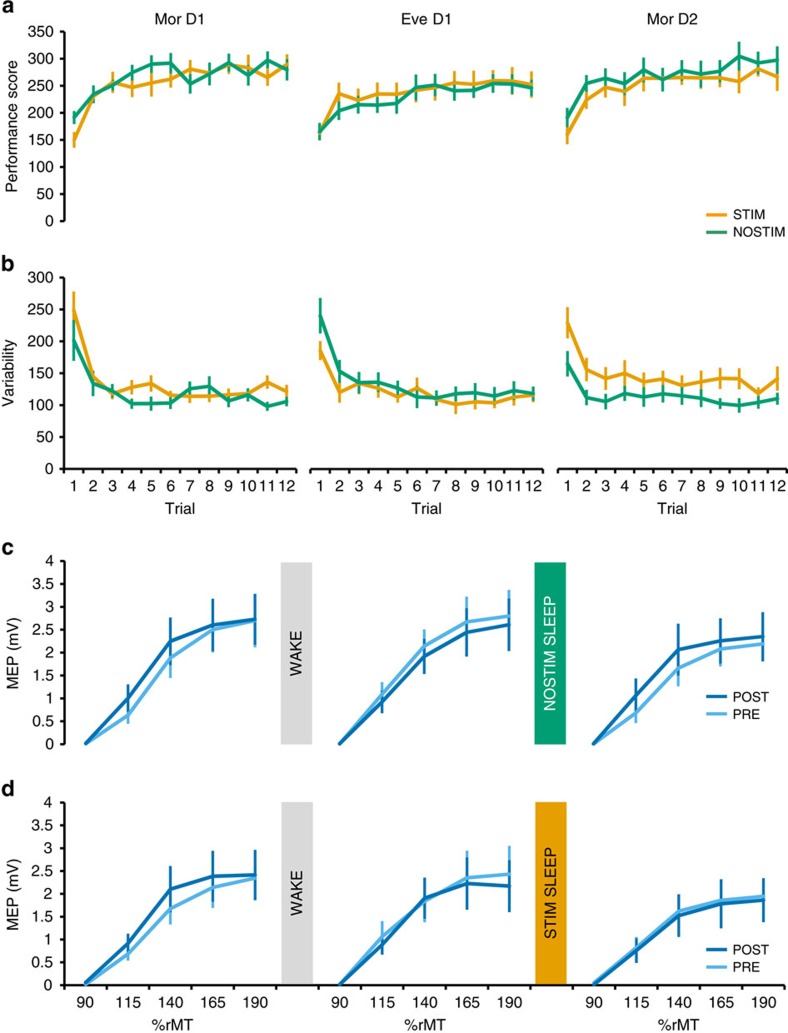
Behavioural and neurophysiological markers. Behavioural and neurophysiological markers of neuroplastic changes in response to motor training. Data (mean±s.e.m.) obtained for each learning assessment (Mor D1, Eve D1, Mor D2) are shown for both the NOSTIM session (green) and the STIM session (yellow) as separate learning curves (**a**) for Performance Scores (% correct sequences divided by inter-tap interval in s, *n*=11) and (**b**) for Variability (average s.d. of inter-tap intervals in completed sequencesn, *n*=11). (**c**,**d**) Input-output curves for the first dorsal interosseous muscle, *n*=10) (see Supplementary Fig. 4 for other intrinsic hand muscles). See Methods for further details.

**Figure 5:**
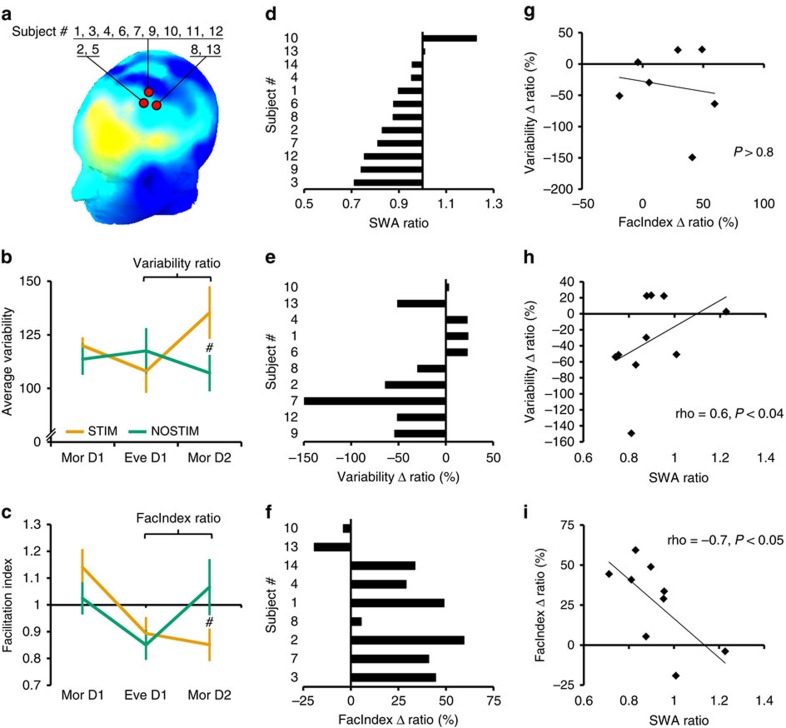
Correlation analysis between low-slow wave activity changes and neoplastic changes in response to motor training. (**a**) Hotspot-electrode locations for each subject depicted on top of the topographical map showing low-SWA differences between the STIM and the NOSTIM sessions (blue colours indicate less SWA during STIM sleep; *n*=13). (**b**) Plateau performance of Tapping Variability (*n*=11) for each learning assessment in both the STIM and NOSTIM session. (**c**) Changes in corticomotor excitability (*n*=10) for each learning assessment were summarized by a Facilitation Index. A FacIndex>1 indicates an increase in corticomotor excitability from PRE to POST (see Fig. 4c,d), while a FacIndex<1 indicates a decrease. (**d**) Rank ordered SWA ratio (SWA STIM/SWA NOSTIM) calculated for the hotspot-electrode of all subjects included in the correlation analysis. Note that small SWA ratios (<1) indicate that less SWA was observed in the STIM than in the NOSTIM session. (**e**) Overnight change in Variability was calculated for all subjects included in the correlation analysis. Note that a Variability Δ ratio<0 indicates an overnight increase in Variability in the STIM compared to NOSTIM session. (**f**) The perturbation related effect on the overnight change in FacIndex was calculated for all subjects included in the correlation analysis. Note that a higher FacIndex Δ ratio indicates a loss in the capacity to exhibit a training-induced increase of corticomotor excitability in the STIM session compared to NOSTIM session. (**g**) There was no significant correlation between Variability Δ ratio and FacIndex Δ ratio (*n*=7). (**h**) The SWA ratio and Variability Δ ratio exhibited a significant positive correlation indicating that a large reduction of SWA during the STIM night was associated with a larger overnight increase in variability when compared to the NOSTIM session (*n*=10). (**i**) The SWA ratio and FacIndex Δ ratio exhibited a significant negative correlation indicating that a large reduction of SWA during the STIM night was associated with smaller increases in corticomotor excitability in response to motor learning (*n*=9). Spearman’s rho coefficient, ^#^*post hoc* analysis STIM vs NOSTIM (*P*<0.03—adjusted alpha level); vertical bars denote s.e.m.s. See Methods section for further details.

Given that a new sequence was performed during each learning assessment (Mor D1, Eve D1, Mor D2) of each experimental session (STIM, NOSTIM), it is not surprising that a significant increase in Performance Score (Fig. 4a) and a significant reduction in Tapping Variability were observed (Fig. 4b) in both STIM and NOSTIM sessions (*training trial* effect for each learning assessment and stimulation session, all *P*<0.001; mixed effects models, *n*=11 after outlier rejection, see Methods section and Supplementary Fig. 1). These learning dynamics were highly similar across learning assessments and on both sessions (three-way stimulation session × learning assessment × training trial interactions all *P*>0.5; two-way stimulation session × training trial interactions all *P*>0.91 for both Performance Score and Tapping Variability; mixed effects models, *n*=11). However, low-SWA perturbation during STIM session sleep significantly decreased the performance score, and increased Tapping Variability when considered across all trials (Performance Score: stimulation session × learning assessment interaction F(2,710)=6.3, *P*=0.002, mixed effects model, *n*=11; Variability F(2,710)=16.08, *P*<0.001, mixed effects model, *n*=11). To further investigate this interaction we analysed plateau performance by collapsing the data across trials 7–12, that is, during the second half of the training blocks when behavioural changes were minor. With this data, no effect of STIM versus NOSTIM was observed for the Performance Score (stimulation session × learning assessment interaction, *P*=0.4; stimulation session main effect, *P*=0.7; mixed effects model, *n*=11), but it was evident that low-SWA perturbation during STIM session sleep significantly increased Tapping Variability on the second morning (Fig. 5b, yellow) in comparison to NOSTIM (Fig. 5b, green; stimulation session × learning assessment interaction F(2,93.9)=3.6, *P*=0.03; mixed effects model, post hoc pairwise analysis between STIM and NOSTIM on second morning, *P*<0.006; *n*=11).

Neural changes associated with motor learning were probed by Transcranial Magnetic Stimulation (TMS) and corticomotor excitability was quantified as the peak-to-peak amplitude of motor evoked responses (MEP). We measured excitability as a function of increasing TMS stimulation intensities (input-output curves (IO curves)) which were acquired PRE and POST-training (Fig. 1c)^14^,^15^. Corticomotor excitability is typically increased (steeper IO curves) after prolonged motor training^14^,^15^,^16^,^17^,^18^ indicating LTP-like plasticity^14^.

Learning a new motor sequence in the morning (Mor D1) led to an increase in corticomotor excitability in the first dorsal interosseous (FDI) as shown by a steeper POST-training IO curve. This increase in excitability from PRE to POST learning was observed for both experimental sessions (Fig. 4c,d; pre–post × intensity interaction for STIM and NOSTIM: *F*(1,63)≥2.97, *P*<0.05; mixed effects model, *n*=10). By contrast, corticomotor excitability decreased from PRE to POST learning when a new sequence was practiced in the evening (Eve D1) (Fig. 4c,d; pre–post × interaction effect for NOSTIM: *F*(1,63)=4.18, *P*=0.01 and STIM: *P*=0.163; mixed effects models, *n*=10). Importantly, the neural response to practicing a new sequence in the morning of day 2 (Mor D2) exhibited again an increase following one night of unperturbed sleep (NOSTIM; Fig. 4c; even though the pre-post–intensity interaction failed to reach significance *F*(1,63)=1.26, *P*=0.29, mixed effects model, *n*=10) while even a slight decrease in excitability on Mor D2 was observed after perturbed sleep (Fig. 4d; *P*=0.99, mixed effects model, *n*=10).

Since the steepness of the IO curve can vary strongly across individuals, we summarized the above changes in IO curves by calculating a Facilitation Index for each learning assessment and experimental session which can then be related to SWA: FacIndex=∫_Intensity 1–5_MEP_post_/∫_Intensity 1–5_MEP_pre_. The FacIndex reflects the normalized change in corticomotor excitability from PRE to POST training and allows us to pool data across the index finger (FDI) and the little finger (abductor digiti minimi (ADM)), that is, two main muscles involved in the motor learning task (Supplementary Fig. 4 shows each muscle separately). We observed that the FacIndex was highest on the first morning and decreased in the evening for both STIM and NOSTIM sessions. However, on the morning of day 2, the FacIndex was only restored after unperturbed sleep, while restoration was markedly reduced after perturbing low-SWA in M1 (Fig. 5c; stimulation session × learning assessment interaction F(2,4537.01)=5.073.88, *P*=<0.01; mixed effects models, post hoc pairwise analysis, *P*<0.01, *n*=10). These interactions remained significant when baseline corticomotor excitability as measured during PRE was added as a covariate of no interest (random effect), indicating that these differences between STIM and NOSTIM do not result from changes in baseline excitability but rather by a diminished capacity of plasticity in response to motor training the next morning.

Next, we investigated whether differences in low-SWA measured under the hotspot-electrode (Fig. 5a) between the STIM and NOSTIM sessions (expressed as SWA ratio=SWA_hotspot_ STIM/SWA_hotspot_ NOSTIM, Fig. 5d) were related to the degree of overnight changes in either motor variability or corticomotor excitability.

We first calculated overnight changes in motor variability (Variability ratio=Var_MorD2_/Var_EveD1_) and corticomotor excitability (FacIndex ratio=FI_MorD2_/FI_EveD1_) (Fig. 5b,c). Then we quantified how much these ratios differed between the STIM and NOSTIM sessions (Variability Δ ratio, FacIndex Δ ratio) and expressed this as a percentage (Fig. 5e,f).

Interestingly, the SWA ratio at the hotspot-electrode was positively correlated with the Variability Δ ratio (Fig. 5h; Spearman’s rho=0.60, *P*<0.04, one tailed; *n*=10 after outlier rejection, see Methods section and Supplementary information 1) indicating that subjects whose SWA was strongly reduced during STIM sleep exhibited elevated levels of motor variability the next morning. Moreover, the SWA ratio at the hotspot-electrode was negatively correlated with the FacIndex Δ ratio (Fig. 5i; Spearman’s rho=−0.7, *P*<0.05; *n*=9 after outlier rejection, see Methods and Supplementary Fig. 1) indicating that subjects whose SWA was strongly reduced during STIM sleep did not recover their capacity to exhibit a training-induced increase in corticomotor excitability overnight. No correlation was found between the Variability Δ ratio and FacIndex Δ ratio (Fig. 5g; *P*>0.05, Spearman’s rho*, n*=7) indicating that behavioural markers of motor learning and neurophysiological markers estimating changes of synaptic strength within M1 circuitry in response to learning are statistically independent phenomena. The lack of a direct association between these markers has been demonstrated before^19^,^20^,^21^,^22^, corroborating the view that changes in corticomotor excitability and behavioural markers of motor learning are two complementary measurements. While behavioural markers are a compound measurement capturing adaptation at multiple levels of the neuromuscular system, corticomotor excitability reflects a subset of the circuits involved in learning with a strong focus on the primary motor cortex^22^. Importantly, our findings indicate that both markers of learning are significantly modulated by SWA.

### Control experiment

**Figure 6:**
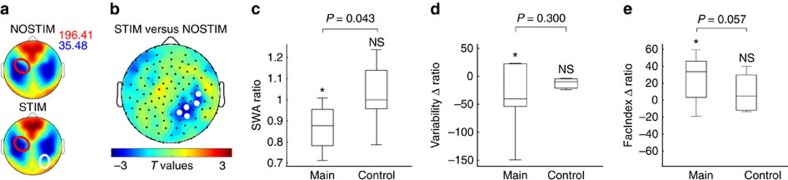
Control experiment and comparison to the main experiment. Control experiment targeting right temporo-parietal cortex (white circle) during STIM nights (*n*=7). (**a**) Topographical map of low-SWA (1–2 Hz) of the two experimental nights scaled to maximum (red) and minimum (blue) power values (μV^2^ per Hz). The red circle indicates the location of the individuals’ TMS hotspot which clearly differed from the electrodes selected for local slow wave perturbation (white circle). (**b**) Statistical comparison (*t* values) of low-SWA between the STIM and NOSTIM sessions (paired *t*-test; *n*=7, white dots, *P*<0.05, uncorrected). Blue colors indicate a decrease and red colors indicate an increase in low-SWA in the STIM compared to the NOSTIM session. (**c**) SWA ratio over the hotspot-electrode (red circle in **a**,**b**) for the main and control experiment. Comparison of the Variability Δ ratio (**d**) and the FacIndex Δ ratio (**e**) between the main experiment and control experiment, using Wilcoxon Rank Sum tests (*P* values above brackets). Box-plots: middle line indicates the median, the bottom and top of the box indicate the first and third quartiles, whiskers extend to 1.5 × the IQR. Stars indicate significant deviations from the value representing ‘no change’ (1 for **c** and 0 for **d**,**e**). See Methods section for further details.

Finally, we conducted a control experiment where subjects (*n*=7) followed the exact same overall procedures but a control electrode overlying right temporo-parietal cortex was targeted during the STIM night. Overall sleep architecture was similar between the two nights except for N2 sleep, which was increased by ∼17.9±5.8 min during the STIM night (paired *t*-test *P*=0.02, *n*=7, Supplementary Table 5). The stimulation caused a significant reduction of SWA by 14.46±4.28% in a cluster of 5 electrodes over the right temporo-parietal area (Fig. 6a,b) while SWA was virtually unchanged at the hotspot-electrode of the left hemisphere (increase of 2.74±5.21%). Thus, applying acoustic stimulation to a control electrode over the right temporo-parietal cortex modulated SWA at the hotspot-electrode significantly less than targeting the hotspot-electrode directly. This finding was further quantified by SWA ratios which did not differ-significantly from 1 and were significantly smaller than when SWA was perturbed at the hotspot-electrode (Wilcoxon rank-sum, 2 groups, *n*=7 and *n*=13, one-sided, *P*=0.04, Fig. 6c). Also in the control experiment during training, a significant increase in Performance Score and a significant reduction in Tapping Variability were observed at all time points (all *P*<0.02, mixed effects models, *n*=7), despite no significant stimulation session x learning assessment interactions for either measure (all *P*>0.5, mixed effects models, *n*=7, Supplementary Fig. 5). Furthermore, mean Variability Δ ratio did not differ significantly from zero (Wilcoxon Sign Rank, *P*=0.3, *n*=7) indicating that perturbing SWA over the right temporo-parietal cortex did not cause an overnight-change in motor variability. In contrast perturbing SWA at the hotspot-electrode (main experiment) caused the Variability Δ ratio to significantly deviate from 0 (Wilcoxon sign-rank, *P*=0.04, *n*=10). Comparing the Variability Δ ratio directly between the main and the control experiment failed to reach significance (Wilcoxon Ranksum one-sided, *P*=0.3, 2 groups, *n*=7 and *n*=10, Fig. 6d), however, perturbing SWA at the hotspot-electrode caused a behavioural effect (Cohen’s d=0.66) approximately twice as large than perturbing SWA in right temporo-parietal cortex (Cohen’s d=0.33). We also analysed the FacIndex for the same muscles as in the main experiment. We scrutinized in particular whether stimulation changed the FacIndex from the evening of day 1 to the morning of day 2 as indicated by the FacIndex Δ ratio, which did not differ significantly from zero (Wilcoxon sign-rank *P*=0.81, *n*=7), and exhibited a very small effect size (Cohen’s *d*=0.03). By contrast, perturbing SWA at the hotspot-electrode had a large effect on FacIndex Δ ratio (Cohen’s d=1.09), which differed significantly from zero (Wilcoxon Sign Rank *P*=0.027, *n*=9). Comparing the FacIndex Δ ratios of the main and control experiments directly, the result was verging upon significance (Wilcoxon Ranksum one-sided, *P*=0.057, 2 groups, *n*=7 and *n*=9, Fig. 6e). Taken together, the results suggest that perturbing SWA under the hotspot-electrode (main experiment) influenced motor plasticity in response to motor training the next morning substantially more than perturbing SWA under the right temporo-parietal cortex (control experiment).

## Discussion

Using a novel approach to perturb slow waves during NREM sleep we demonstrated that a reduction of SWA in M1 causally influenced the capacity to undergo motor learning and adaptive brain responses to motor training the next morning, such that larger reductions in SWA due to our experimental manipulation were associated with less efficient adaption at the behavioural and neural level. Interestingly, perturbing SWA in M1 had little influence on average performance gains in response to training, however, it significantly increased tapping variability which remained elevated after reaching plateau performance and it prevented a training-induced increase in corticomotor excitability that is otherwise observed in response to motor training.

This finding in healthy human volunteers was revealed using a non-invasive approach with the defining feature that acoustic stimulation was triggered by slow waves detected by the EEG electrode that was closest to the FDI hotspot, timed such that the slow waves returned more rapidly to the positive up-phase when compared to unperturbed sleep (Fig. 3a,b). As a consequence large amplitude slow waves occurred less frequently (Fig. 3d) and this effect was only observed for electrodes close to the target area, with a tendency to spread to the back of the head. Since slow waves are not phase locked across different brain areas^23^, our paradigm caused these specific local effects because slow waves in M1 were consistently stimulated during the negative phase, while slow waves in other areas were most likely stimulated at random phases (for topographical slow wave detection distribution see Supplementary Fig. 6). However, slow waves are not stationary either. The majority originate in the frontal cortex and travel backwards^23^. Therefore, we quantified the travelling of slow waves in an exploratory analysis (using the toolbox presented in Mensen *et al*.^24^). Slow waves, travelling through the hotspot electrode, passed significantly less electrodes located posterior from the hotspot-electrode in the STIM night compared to the NOSTIM night. Thus, by interacting with the ongoing slow oscillation at the hotspot-electrode the travelling of the slow waves might be interrupted, which might explain the reduction of low SWA posterior from the hotspot-electrode. The precise interaction of local slow wave disturbance and the consequences on slow wave travelling needs to be elaborated in more detail in future studies.

Moreover, the general sleep architecture was very similar between the two sessions, except for a tendency toward reduced N3 sleep during STIM night (mean difference 12.7±5.6 min). However, the difference in N3 sleep was not related to the impaired recovery process of cortical excitability nor the reduced improvement in variability (VAR: Spearman’s rho=0.2, *P*=0.58, FacInd: Spearmans’s rho=−0.17, *P*=0.68). In addition, psychological measurements (that is, motivation, mood, attention etc. assessed by questionnaires) were also very similar across sessions, illustrating that the perturbation was highly specific to low-SWA in M1 (Supplementary Discussion) and that our behavioural and neural measurements were unlikely confounded by lack of attention, motivation or other side effects typically associated with general sleep deprivation. Of course, our viewpoint from the surface of the scalp provides only an indirect measure of brain activity, as we detect only large population neuronal oscillatory activity. Nevertheless, using this completely non-invasive method we provide the first evidence that local deprivation of sleep slow waves can be achieved in humans. The value of this tool is further increased by the recent observation that a similar approach also serves to boost slow waves^25^. As such, this simple and non-invasive approach allowing long-term stimulation is also ideally suited for clinical translation, for example, benefitting new therapeutic approaches that take advantage of sleep to modulate specifically affected brain areas in a way that will not affect daytime performance.

Motor training was tested using different motor sequences because this task is well-suited to repeatedly probe the effect of motor training^26^. During all learning assessments motor sequence production was optimized by repetitive training, which resulted in more accurate (Fig. 4a) and less variable motor execution (Fig. 4b). Even though this finding might be surprising at first, one has to keep in mind that we changed SWA in M1 only by approximately 13%. Thus, similar to the recent observation by Lustenberger *et al*.^27^ one would expect that our experimental manipulation causes relatively small behavioural effects which might be most strongly reflected by temporal aspects of task performance. We found that perturbing SWA in M1 during sleep resulted in a general increase in tapping variability the next morning that was significantly elevated when compared to performance after unperturbed sleep (Figs 4b and 5b). Thus, even though the subjects improved their mean performance, motor execution was generally less efficient. The benefit of local deep sleep for motor behaviour may be to keep baseline motor variability low to assure optimality of motor control and learning^28^, a result in line with previous evidence indicating that sleep reduces variability of both postsynaptic firing and behaviour via a down-scaling mechanism^29^,^30^.

We further demonstrated that sleep modulated neurophysiological responses to motor training. Motor training has been shown to strengthen input-output relationships of the activated motor circuits (that is, the same TMS input evokes a larger physiological response after training) most likely by potentiating synapses via a LTP-like mechanism^31^,^32^,^33^,^34^. We show that the brain’s capacity to exhibit synaptic potentiation is high in the morning but significantly reduced in the evening (Figs 4c,d and 5c), lending further support to the hypothesis that synaptic plasticity gets saturated during long periods of wakefulness^26^,^35^. One night of unperturbed sleep increased the brain’s capacity to exhibit synaptic potentiation the next morning, however, locally perturbing SWA in motor cortex was sufficient to diminish this effect (Fig. 5c,i). A control experiment further confirmed that overnight changes in the ability to effectively reduce motor variability due to practice as well as changes in the neurophysiological response to motor training were much less influenced when SWA was perturbed in a control region (that is, right temporo-parietal cortex). Thus, local deep sleep appears to be mechanistically involved in re-normalizing synaptic potentiation overnight, thus ensuring that the brain’s capacity to efficiently adapt to the environment via plastic changes at the synaptic level is restored on a day-to-day basis.

Our stringent statistical outlier detection procedures resulted in low sample sizes for some analyses containing only a sub-set of participants, and as such, those results should be interpreted as preliminary and warranting future replication. Nevertheless, it is clear that our method for modulating deep sleep is promising and that effectiveness might be further enhanced by more precise timing of the tones relative to the phase of slow waves as shown in Supplementary Fig. 7. As such we introduce a new tool to shed further light upon important concepts regarding the biological function of sleep and to provide additional evidence that there is a causal link between SWA while sleeping and re-normalization of neural processes that restore the capacity of plasticity in response to motor training. Taken together our findings consistently indicate that local deep sleep is essential for maintaining the brain’s capacity to respond efficiently to motor training and thus for adapting to the environment.

## Methods

### Subjects participating in the main experiment

31 naive (no prior experience with the motor task, no musicians) healthy right-handed (94.3±9.9%; (ref. 36)) subjects (13 females, mean±s.e.m. age; 22.02±0.34 years) complied with the following inclusion criteria and participated in the main experiment: No personal or family history of psychopathology, no severe brain injury, no sleep disorders, no chronic diseases, no current use of psychoactive agents or other medications, no travelling across more than 1 time zone in the 4 months before the study, no previous adverse reactions to Transcranial Magnetic Stimulation (TMS), a resting motor threshold (rMT) exceeding 50% of the stimulator output (as tested during a familiarization session, see below). The latter criterion was necessary to protect the TMS equipment from over-heating since our procedure required repeated stimulations of up to 190% rMT. Written informed consent was obtained prior to participation. The study was approved by the local ethics committee and performed according to the Declaration of Helsinki.

Of the 31 subjects that complied with the inclusion criteria, 16 dropped out for the following reasons: discomfort caused by TMS (*n*=1), poor sleep during screening night (*n*=9), not sticking to a regular sleep wake cycle or not tolerating the auditory stimulation (*n*=6; for a flow chart of subject dropouts see Supplementary Fig. 1, upper part).

### Behavioural task

Subjects performed a computerized six-element finger sequence tapping task (presented with E-Prime; Psychology Software Tools, Inc., Sharpsburg, USA) adapted from Karni *et al*.^37^. The sequence to be executed was depicted on top of the laptop screen using a numbering system, with 1, 2, 3, and 4 corresponding to the index, middle, ring and little fingers of the right hand respectively (Fig. 1c). Throughout the experiment eight equally difficult sequences were used. None of these sequences were trained twice and the order of sequences used throughout the experiment was randomized across subjects. While tapping the sequence a black dot appeared on the screen below the current number every time the subject pressed a key indicating that a response was recorded without giving any accuracy feedback. When a sequence was completed the screen was refreshed so that the same sequence appeared on top without any black dots present. One training trial consisted of typing the given sequence for 30 s as many times as possible followed by a rest period of 30 s to prevent fatigue. A training block consisted of 12 consecutive trials.

### Electromyography (EMG) and TMS

Focal TMS was applied with a 70 mm figure-of-eight coil connected to a Magstim 200 stimulator (Magstim, Whitland, Dyfed, UK). The coil was positioned over the M1 of the left hemisphere, tangential to the scalp with the handle pointing backwards and laterally at 45° away from the mid-sagittal line^38^. The optimal scalp position (‘hotspot’) for stimulating the right first dorsal interosseous (FDI) was identified and marked for each learning assessment. The rest motor threshold (rMT, lowest stimulus intensity evoking MEPs with amplitudes of at least 50 μV in 5 out of 10 consecutive stimuli) was determined prioritizing the FDI^39^.

EMG data was recorded from FDI, abductor digiti minimi (ADM) and opponens pollicis (OP) of the right hand (Bagnoli, Delsys, Inc., Natick, USA) (for an overview of detailed results for all three muscles see Supplementary Fig. 4). The signals were sampled at 5000 Hz (CED Power 1401, Cambridge Electronic Design, UK), amplified, band-pass filtered (5–1,000 Hz), and stored on a PC for offline analysis. Pre-stimulus EMG recordings were used to assess the presence of unwanted background EMG activity in the 110 to 10ms time interval preceding the magnetic pulse.

Corticomotor excitability was quantified by measuring input-output curves (IO curve) using 90, 115, 140, 165 and 190% of rMT. One IO curve consisted of 20 MEPs per intensity. They were acquired in 2 blocks of 50 MEPs so that per block 10 stimulations were acquired for each of the 5 intensities. In between blocks a rest period of ∼30 s was provided. Within one block the inter-stimulation interval varied between 6 and 7 s resulting in a total block time of 5 min.

### High-density sleep electroencephalography

All night sleep was recorded, referenced to the vertex (Cz), sampled at 500 Hz (0.01–200 Hz), using high density (hd) EEG (Electrical Geodesics Sensor Net for long-term monitoring, 128 channels, Electrical Geodesics Inc., EGI, Eugene, OR, USA). Electrooculographic (EOG) and submental EMG recordings were acquired for visual scoring of sleep. Two additional electrodes (gold, Grass Technologies, West Warwick, RI, USA) were attached to the earlobes, which served as reference electrodes for the online slow wave detection (see ‘SWA topography and sleep slow waves analysis’). After adjusting the net to the vertex and the mastoids, all electrodes were filled with an electrolyte gel to ensure the maintenance of good signals throughout the night. In general, electrode impedances were below 50 kΩ. Impedances were below 20 kΩ for the electrodes used for online slow wave detection; that is, submental electrodes, electrodes on the earlobes, and the hotspot-electrode defined as the EEG electrode closest to the scalp position of the FDI hotspot as previously determined by TMS.

### Real time closed-loop slow wave detection

Using a custom LabVIEW (National Instruments, Austin, TX, US) program, a closed-loop algorithm detected sleep slow waves in real time (loop time ∼30 ms) and administered acoustic stimuli to selectively modulate sleep slow waves based on the EEG electrode that was closest to the FDI defined as TMS hotspot-electrode as determined by TMS (Fig. 1b,c). For all subjects the TMS hotspot-electrode was one of the following channels: CH29, CH30 or CH36 within the EEG net. The EEG signal of the TMS hotspot-electrode was re-referenced to the mean value of the earlobe electrodes and the signal was band-pass filtered (Butterworth 0.5–2 Hz, stop-band<0.1 and >10 Hz, stopband attenuation 20 dB, passband attenuation 0.1 dB). In parallel the submental EMG was monitored, by continuously calculating the root mean square over 2 s. Every time the loop was turned on, tones (pink 1/*f* noise of ∼50 dB) were played for precisely 50ms whenever the EEG signal crossed a default threshold (for the main experiment −30 μV, for the control experiment −25) and the EMG was below a given threshold that was continuously monitored and adapted by the experimenter. The EMG threshold was implemented as a safety component to prevent stimulation while there were any signs of arousal. The manual stimulation procedure conducted by the experimenter was the following: Stimulation started after 10–15 min of stable N3 sleep. The online slow wave detection algorithm was turned on throughout the night but only during stable NREM sleep stages 2 and 3 (appearance of spindles, K-complexes and slow waves). The goal was to stimulate as much as possible without waking the subjects (on average 3,143.69±354.73 stimuli were applied, Supplementary Table 4). It is important to mention that the overall goal of the study was only to locally disturb slow wave sleep, without interfering with the global structure of sleep. Therefore, the online slow wave detection algorithm was turned off during each transition and after every sign of arousal (that is, increase in EMG or break down of the EEG) and kept off until the EEG displayed stable N2 or N3 sleep again. As a consequence, tones were applied to only ∼50% of all slow waves.

Offline verification of the online slow wave detection algorithm was performed by measuring the instantaneous phase of each slow wave at tone onset. To do so, the EEG signal of the TMS hotspot-electrode was re-referenced to the mean signal from the two earlobes and band-pass filtered between 0.5 and 2 Hz (using zero-phase infinite impulse response Butterworth filter with the same filter settings as for online slow wave detection, stop-band <0.1 and >10 Hz), followed by a Hilbert transform to define instantaneous phase. Phase angles (0–360°) were defined with 0° corresponding to the negative peak of the slow wave cycle. Percentage of tones applied during the negative phase of the slow wave cycle (between 270°–360° corresponding to the first negative half wave or between 0°–90° corresponding to the second negative half wave), were calculated (for offline analysis of phase timing of tone onset and local slow wave perturbation see Supplementary Fig. 7).

### Overall experimental protocol

Subjects participated in three sessions: (i) a screening and familiarization session for TMS and sleeping with an EEG net in the laboratory; (ii) a first experimental session; and (iii) a second experimental session 1 week later.

*TMS screening and familiarization session*. After the subject was screened for TMS exclusion criteria and possible adverse effects by the experimenter, he/she was seated in a comfortable chair with the right forearm resting in a neutral position while the hotspot and rMT of the right FDI were determined (see ‘EMG and TMS’). Subjects with a rMT exceeding 50% maximal stimulator output were excluded from further participation since the study design required stimulations up to 190% rMT. For subjects with a sufficiently low rMT, one IO curve was obtained in order to familiarize subjects with this measurement technique. Subjects that indicated discomfort during the measurement or reported particular after-effects of the stimulation (for example, headache) were excluded from further participation. After the corticomotor excitability measurement, subjects sat in front of a laptop on which they performed the behavioural task as it would appear during the experimental sessions. They were instructed to tap a given six-element sequence, that is, either FAM1 or FAM2 (balanced amongst subjects) as quickly and accurately as possible for 12 training trials of 30 s (see ‘Behavioural task’). Note that sequences used during familiarization were not re-used during any of the experimental sessions.

*Sleep screening and familiarization session*. Prior to the experimental session, subjects spent one night in the sleep laboratory of the University Children’s Hospital Zurich (Zurich, CH) wearing the hd EEG net in order to adjust to the experimental environment and to exclude possible sleep disorders. Sleep quality was assessed, and only subjects with a sleep efficiency of at least 80% were included in the experiment.

*Experimental sessions*. After successfully participating in both TMS and EEG screening sessions, subjects were instructed to keep a constant sleep schedule for ∼7 days (range from 4–8 days) preceding the first experimental session. Compliance to the schedule was assessed using daily sleep diaries and wrist actigraphy (Actiwatch Type AWL from Cambridge Neurotechnology, CamNtech, Cambridge, UK or Geneactiv). Subjects were required to refrain from alcohol and medication 48 h prior to each experimental session. During the experimental sessions, subjects did not perform strenuous exercise, and were not allowed to nap during the day (as instructed and verified via self-report and actigraphy; for analysis of wrist actigraphy see Supplementary Table 6). Women were tested during the follicular phase of their menstruation cycle to prevent the variation of sleep EEG activity markers as a consequence of hormonal fluctuations^40^.

Subjects were tested in two experimental sessions that were separated by 1 week but had an identical chronological setup of three separate learning assessments; the first in the morning of day 1 (Mor D1), the second in the evening on that same day (Eve D1), and a third in the morning the next day (Mor D2, Fig. 1a). In one of the two experimental sessions, slow waves were perturbed using acoustic stimulation (STIM) precisely time-locked to the down-phase of sleep slow waves detected in the TMS hotspot-electrode (Fig. 1b). In the other experimental session subjects heard no tones (NOSTIM). The order of the STIM versus NOSTIM experimental session was counterbalanced among subjects (six subjects started with STIM).

The first learning assessment started (approximately) 30 min after awakening in the morning and subjects filled out a questionnaire rating the sleep quality of the night before (that is, assessing subjective sleep score with a visual analog scale) and a questionnaire rating psychological measurement (that is, attention, mood, concentration and motivation, also on a visual analog scale, which was assessed before each learning session, that is, in the evening and the next morning). The hotspot and rMT for the right FDI were then determined and corticomotor excitability was measured via an IO curve (PRE). Subsequently subjects performed motor training, that is, they practiced a new six-element motor sequence (for example, sequence A) for 12 trials (that is, 30 s tapping followed by 30 s rest) lasting 12 min in total. Corticomotor excitability was re-measured immediately afterwards by a second IO curve (POST) in order to determine changes indicative of motor plasticity as a consequence of training (Fig. 1c). It has been shown that repetitive motor training leads to an increase of corticomotor excitability, an effect that is NMDA receptor dependent, sensitive to GABAergic inhibition^32^, and it occludes subsequent experimental induction of long-term-potentiation^14^,^34^,^41^. Together these findings strongly suggest that increases of corticomotor excitability in response to motor training reflect, at least partly, a LTP-like mechanism at the synaptic level activated by extensive motor practice and associated motor learning^14^,^32^,^34^.

The second learning assessment on the evening of the same day started ∼2 h 30 min before bedtime. Subjects underwent an identical procedure as during the morning, with the only difference being a novel finger sequence was trained (for example, sequence B). Immediately afterwards the subjects were prepared for EEG measurements. Before subjects went to bed they were asked to perform a short attention task, based on an auditory oddball paradigm. During the task, participants had to sit quiet in front of a screen and fix a white cross on a black background. Over a period of ∼6 min participants listened to 450 stimuli with an inter-stimuli interval of 0.8 s, whereof 90% were standard tones (880 Hz) and 20% were deviant tones (988 Hz, presented in a random order). Subjects were asked to react as fast as possible to the deviant tones with a mouse click. Reaction time to the deviant tones, number of missed clicks and number of wrong clicks were recorded as objective vigilance scores. After the attention task subjects went to bed at their usual bedtime. Sleep episodes were scheduled individually according to the subject’s reported bed times with a sleep time of ∼8 h.

In the morning of the next day, subjects were awakened by the experimenter. After performing the same attention task as in the evening the EEG net was removed and a shower was taken within 30 min before starting the third learning assessment. This test was timed so that learning the novel sequence (for example, sequence C) took place 24 h after acquiring the first sequence on the morning of the first day. Identical to the previous learning assessment performed on the previous morning, subjects filled out a questionnaire regarding their quality of sleep the night before and the questionnaire rating psychological measurement, after which corticomotor excitability was tested PRE and POST sequence tapping.

This complete experimental session combined three learning assessments that spanned over 2 consecutive days including one night in the sleep laboratory. It was repeated 7 days later and subjects were therefore instructed to keep the same constant sleep schedule for the following 7 days preceding the second experimental session, which was again assessed using daily sleep diaries and wrist actigraphy. The second experimental session followed an identical protocol to the first one with the only difference being the sleep slow wave intervention (that is, STIM versus NOSTIM) and that three new sequences were learned (for example, sequences D, E, F) during the three learning assessments.

Assignment of sequences to experimental conditions and learning assessments was randomized and counter balanced among subjects.

### Outlier detection and removal for the main Experiment

We aimed for a good balance between keeping the sample size for each analysis as high as possible whilst applying a stringent outlier detection strategy which we defined a-priori and applied consistently to all parameters. Outlier detection and removal is particularly important to minimize the risk of bias due to uncontrolled confounding factors. For example, we were very strict to include only participants that had high-quality sleep in both the STIM and the NOSTIM night. Otherwise our data might have been confounded by large differences in the general sleep architecture.

For each parameter, we calculated the mean and standard deviation across all participants available for the specific comparison (that is, with potential outliers included in the dataset). An individual’s data were identified as outlier when it fell outside of the mean±2 × s.d. range and this individual was then removed from the statistical analysis. Outlier detection and removal was performed stepwise as detailed in Supplementary Fig. 1, lower part). The final analyses were performed on the following sample sizes: SWA analysis (*n*=13), performance index and variability (*n*=11), correlation between SWA ratio and Variability Δ ratio (*n=*10), FacIndex analysis (*n*=10), correlation between SWA ratio and FacIndex Δ ratio (*n*=9).

### Data analysis and statistics

Inferential statistics were computed using Mixed Effects Models in SPSS (Version 16.0, SPSS Inc. Chicago, US), as they account for covariances between related data samples in repeated measures designs, and have greater flexibility for modelling effects over time than traditional ANOVA approaches^42^. Moreover, they use all available data, allowing inferences to be made even from small sample sizes, and they do not assume normality of the raw data^43^. Fitting of the mixed effects models employed restricted maximum likelihood estimation (REML) and a compound symmetry or unstructured covariance matrix. Model fit indices (Akaike Information Criterion and Schwarz Bayesian Criterion) were considered prior to choosing the covariance matrix and model type. The influence of each of the fixed effects on the model was estimated using *F* tests. In all models *subject* was designated as a random effect with random intercepts.

*Behavioural task*. Key presses were recorded and accuracy (%) was calculated as the number of correct sequences divided by all completed sequences during each 30 s trial. Performance *speed* was measured as the time (s) between key presses, that is, the inter-tap interval (ITI). A *Performance Score* was calculated for each subject and trial by dividing the accuracy percentage by the ITI, with higher scores indicating better performance (see also^44^). Tapping Variability was determined by calculating the standard deviation in ITI per typed sequence and was then averaged for each 30 s trial and subject. Plateau performance was estimated by averaging the Performance Score and Variability across trial 7–12, that is, during the second half of the training blocks when behavioural changes were minor.

Mixed effects models were performed on both the Performance Score and Variability data with repeated fixed effects of stimulation session (STIM, NOSTIM), learning assessment (MorD1, EveD1, MorD2) and training trial (1–12). Plateau Performance Score and Variability data were analysed with an analogous model containing the fixed effects stimulation session and learning assessment.

*Corticomotor excitability*. Corticomotor excitability was quantified by MEP peak-to-peak amplitude. MEP amplitude is known to be modulated by EMG background activation^45^,^46^. Therefore pre-stimulus EMG recordings were used to assess the presence of unwanted background EMG activity in the 110 to 10ms preceding the magnetic pulse and were quantified via root mean square scores (RMS) across this interval. The maximal and minimal MEPs obtained per intensity and per IO curve were excluded as well as the obtained MEPs preceded by background EMG higher than 0.01mV. For each subject and over all trials we calculated the mean and standard deviations of the background EMG. Background EMG values deviating from the mean by more than 2.5 standard deviations, and MEPs with a peak-to-peak amplitude which exceeded Q3+1.5 × (Q3−Q1) were removed from further analysis, with Q1 denoting the first quartile and Q3 the third quartile computed over the whole set of trials for each subject. Based on these criteria 15.2±2.3% of the collected MEPs were excluded from further analyses. Furthermore we averaged MEP amplitudes for each stimulation intensity of each IO curve that was recorded and these averages where then subjected to group statistics.

The influence of motor training on corticomotor excitability as quantified by the IO curve was analysed using a mixed effects model with repeated fixed effects stimulation session (STIM, NOSTIM), learning assessment (MorD1, EveD1, MorD2), pre-post (PRE, POST) and intensity (90, 115, 140, 165, 190%). Changes in baseline corticomotor excitability (that is, PRE IO curve measured prior to motor training) across learning assessments were tested for each of the two experimental sessions by separate mixed effects models with the factors learning assessment (MorD1, EveD1, MorD2) and intensity (90, 115, 140, 165, 190%).

Finally we directly compared whether changes in corticomotor excitability induced by motor training differed between sessions and learning assessments. Therefore, we calculated the integral underneath the IO curve measured before and after motor training^47^, and calculated a Facilitation Index, FacIndex=∫_Intensity 1–5_MEP_post_/∫_Intensity 1–5_MEP_pre_.

A FacIndex>1 indicates that an increase in corticomotor excitability is observed from PRE to POST training, while a FacIndex<1 represents a decrease. The FacIndex was calculated for the 2 stimulation sessions and for each of the 3 learning assessments and was entered into a mixed effects model with repeated fixed effects stimulation session (STIM, NOSTIM) and learning assessment (MorD1, EveD1, MorD2).

*Slow wave activity topography and sleep slow waves analysis*. Sleep EEG was band-pass filtered between 0.5 and 50 Hz and down sampled to 128 Hz. Sleep stages and arousals were visually scored for 20 s epochs according to standard criteria^48^ by a sleep expert, and verified by another sleep expert (both of them were blind to the experimental conditions). After visual and semiautomatic artefact removal^49^ the remaining data of each subject were re-referenced to an average value across all 109 channels above the ears. In one subject only the first 4 h of the night could be analysed due to poor data quality for the remaining part (subject #2). Spectral analysis (1–25 Hz divided into different frequency ranges: low-SWA (1–2 Hz), SWA (1–4.5 Hz), theta (4.75–7.75 Hz), alpha (8–9.75 Hz), sigma (10–15 Hz) and beta (20–25 Hz), for a detailed overview of the results see Supplementary Fig. 3 and Supplementary Table 7) of consecutive 20 s epochs (FFT routine, Hamming window, averages of five 4 s epochs, resolution of 0.25 Hz) of each channel was performed and data were log-transformed for parametrical statistical analysis. Comparisons between STIM and NOSTIM sessions were performed for an individual hotspot-electrode (that is, the EEG electrode showing the strongest reduction of low SWA during STIM night out of the three selected TMS hotspot-electrodes (CH30, CH29 and CH36 of the HCG sensor Net 128 of EGI) to account for anatomical differences related to the combined MEP of ADM and FDI as done previously, for example, in^11^, as well as for each EEG channel.

Further analysis based on single sleep slow waves was performed for the individual hotspot-electrodes. For both sessions, the EEG signal (sampling rate 500 Hz) of the hotspot-electrode was offline re-referenced to the mean value of the earlobe and filtered, using the same filter as for the online detection (Butterworth 0.5–2 Hz (stop-band <0.1 and >10 Hz)).

In a first step the immediate response to the acoustic stimulation was analysed. Therefore the following procedure was applied:

1. For both nights, sleep slow waves were detected offline (similar to the online algorithm applied during STIM session). A trigger was set, every time the signal crossed a default threshold of −30 μV, in all artifact free N2 and N3 epochs.
2. All detected slow waves slower than 0.5 Hz were excluded to ensure that the signal is not influenced by the filter.
3. To account for overall changes in slow wave characteristics (in particular the slope)^50^ we controlled for the number of detected slow waves across the night. To do so, individual sleep cycles were defined for both nights and only sleep cycles including at least 100 triggers during both nights were considered for the analysis.
4. For the NOSTIM session the timing of the first trigger was individually matched to the timing of the first trigger of the STIM session (after 10–15 min of stable N3 sleep).
5. For the STIM session only triggers where a tone was presented during the night were included. During the STIM session we aimed to stimulate as much as possible without waking up the subjects. As a consequence the stimulation algorithm was turned off by the experimenter if there was any sign of arousal, or during the cycle transitions (from NREM to REM or vice versa). Thus, some slow waves were missed during the STIM session, where no tones were played. Hence, less slow waves were detected during the STIM compared to the NOSTIM session. To account for this difference in the number of detected slow waves, we matched the number of analysed waves between the two nights. To do so, for each sleep cycle, equal amounts of detected slow waves during the STIM session were randomly selected in the NOSTIM session. Next, the immediate peak-to-peak up-slope following the stimulation (dividing peak-to-peak amplitude by the time between negative and positive amplitude) and the duration of the slow waves were calculated (time between the negative zero-crossing (start of the down-phase) prior to stimulation and the next negative zero-crossing (end of the up-phase)). This approach led to similar results as calculating the median peak-to-peak up-slope and duration of slow waves, including all detected slow waves. Since the former approach always results in slightly different numbers, the median peak-to-peak up-slope and duration of slow waves were taken for further statistics.

In a second step the induced changes of slow wave characteristics throughout the night (that is, not only including slow waves which were directly disturbed) were analysed. For this the following procedure was applied:

1. All waves starting from the zero crossing of the up-phase, that is, the positive part of the wave, and ending at the second zero crossing of the next down-phase of any amplitude between 0.5 and 2 Hz during N2 and N3 sleep were detected offline, using a similar routine as described by Riedner *et al*. 50.
2. From these detected waves the mean peak-to-peak down-slope (dividing peak-to-peak amplitude by the time between positive and negative amplitude) for each subject and night were calculated.
3. Moreover, the probability of slow waves (defined as slow waves with a peak-to-peak amplitude>75 μV) was calculated by dividing the number of detected slow waves (>75 μV) by the total number of detected waves. For exploratory purposes the analysis of induced changes on general slow wave characteristics was extended to all channels (for this analysis data were down sampled to 128 Hz to reduce computational load).

Since all sleep data were normally distributed (normality of the data was confirmed using Shapiro-Wilk-Test), paired *t*-tests (two-tailed) were used for intra-individual comparisons. For topographical comparisons nonparametric cluster-based statistical testing was applied, using a suprathreshold cluster analysis to control for multiple comparisons^11^,^51^,^52^. In short: For each topographical statistical analysis (paired Student’s *t*-test and Spearman’s correlation) new datasets were generated by randomly relabelling the condition label from original data and either paired Student’s *t*-test or spearman correlations were performed, respectively. For each permutation the maximal size of resulting clusters with neighboring electrodes reaching a *t*-value above the critical value was counted to build a cluster size distribution. From this cluster size distribution the 95th percentile was defined as critical cluster size threshold. For the true comparison (STIM vs. NOSTIM) only electrodes reaching a *t*-value beyond the CV and located within a cluster larger than the critical cluster size threshold were considered as significant (paired Student’s *t*-test: CV=2.3, number of permutations 5000, *n*=13, spearman correlations: CV=0.68, number of permutations 1,000, *n*=9 and CV=0.6, number of permutations 1000, *n*=10). All offline analyses of the sleep data and statistical analyses were performed in MATLAB (MathWorks).

*Correlation analysis*. We calculated a SWA ratio (SWA_hotspot_ STIM/SWA_hotspot_ NOSTIM) representing the change in SWA measured at the hotspot-electrode between the STIM and NOSTIM sessions. Note that a SWA ratio<1 indicates reduced SWA at the hotspot-electrode during the STIM compared to the NOSTIM condition.

We also calculated a ratio of overnight change in variability (Variability ratio) by dividing the Variability data obtained in the morning on day 2 by the Variability of the evening on day 1 (Var_MorD2_/Var_EveD1_). This was conducted separately for the STIM and NOSTIM sessions. Then, the effect of perturbation was quantified by expressing the difference between the STIM and NOSTIM ratio as a percentage of NOSTIM (Variability Δ ratio). This percentage represents the perturbation related effect on the overnight change in Variability.


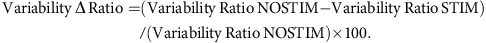


Note that a lower Variability Δ ratio indicates an increase in Variability in the STIM session compared to the NOSTIM session.

Identical calculations were performed on the FacIndex data. We calculated a ratio of overnight change in FacIndex (FacIndex ratio) by dividing the FacIndex calculated for the morning on day 2 by the FacIndex calculated for the evening on day 1 (FI_MorD2_/FI_EveD1_). This was conducted separately for the STIM and NOSTIM sessions. Then, the effect of perturbation was quantified by expressing the difference between the STIM and NOSTIM ratio as a percentage of NOSTIM (FacIndex Δ ratio). This percentage represents the perturbation related effect on the overnight change in FacIndex.


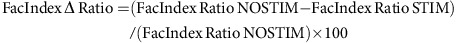


Note that a higher FacIndex Δ ratio indicates a loss in capacity to exhibit a learning-induced increase in corticomotor excitability in the STIM session compared to the NOSTIM session.

To explore whether changes in SWA caused by STIM versus NOSTIM were statistically related to overnight changes in behavioural and neural markers of plasticity, Spearman’s rank correlation coefficients were calculated between (i) SWA ratio and Variability Δ ratio and (ii) SWA ratio and FacIndex Δ ratio.

These analyses used low-SWA (1–2 Hz) measured at the hotspot-electrode, however, control analyses were conducted for all other frequency ranges and at all 128 electrodes (Supplementary Fig. 3 and Supplementary Fig. 7).

The alpha level for all statistical tests was set initially to 0.05 and significant interactions were further analysed by post hoc tests, corrected for multiple comparisons in line with the Modified Bonferroni procedure^53^, resulting in an adjusted alpha value of 0.03. Null hypotheses were tested two-sided unless otherwise stated. Data are presented as mean±s.e.m.

### Control experiment

14 subjects complied with the inclusion criteria but 7 dropped out due to poor sleep during screening night (*n*=4), not sticking to a regular sleep wake cycle or not tolerating the auditory stimulation (*n*=3). Seven naïve, healthy, right-handed (95.4±6.2%; ref. 36) subjects (23.76±1.03 years, 4 females) completed the control experiment. The control experiment followed the exact same experimental procedure as the main experiment, only that slow waves were perturbed using acoustic stimulation (STIM) precisely time-locked to the down-phase of sleep slow waves detected at an electrode overlying the temporo-parietal cortex of the right hemisphere (that is, the hemisphere ipsilateral to the moving hand). Because the mean amplitude of slow waves is generally lower over the temporo-parietal region compared to the TMS hotspot region (that is, primary motor cortex) we also adapted the threshold for online slow wave detection slightly, to ensure that in the control experiment the same number of slow waves were detected as in the main experiment. To do so we calculated the mean amplitude over the temporo-parietal electrode (control electrode) and the hotspot-electrode of the main experiment. We found that the mean amplitude was reduced by ∼20% over the temporo-parietal cortex compared to the hotspot-electrode. As a consequence we reduced the amplitude threshold from 30 to 25 μV for the control experiment. The electrode over temporo-parietal cortex was selected because the right temporo-parietal area is believed to have only a minor contribution to the sequence learning task and previous experiments from our lab have indicated that coherence in the SWA frequency range was low between the hotspot-electrode and the control electrode over right temporo-parietal cortex, indicating that slow waves occur independently in the two areas.

*Outlier detection, data analysis and statistics for the control experiment*. We calculated the same parameters as above. No outliers were detected when we applied the same cutoff-criteria as in the main experiment. Our statistical analysis focused on the Variability Δ ratio and the FacIndex Δ ratio because these parameters summarize how STIM versus NOSTIM influences changes of behavioural and electrophysiological learning indices between the evening of Day 1 and the morning of Day 2. Since the sample size of the control group (*n*=7) and the sample size of the experimental group (*n*=13) was relatively low we applied non-parameteric statistics. We used (i) the Wilcoxon sign-rank test to probe whether STIM induced SWA reduction, Variability Δ ratio or the FacIndex Δ ratio differed significantly from zero for either the experimental or the control group; and ii) the Wilcoxon rank-sum test for probing whether STIM induced SWA reduction, Variability Δ ratio or the FacIndex Δ ratio differed significantly between the main and the control experiment. Since we had a clear directional hypothesis the Wilcoxon rank-sum test was one-sided. Additionally, we calculated Cohen’s *d* effect sizes for each of the above parameters and each group.

### Data availability

The ethical approval granted to the authors by the IRB does not allow the publication of the raw data online. If readers would like to re-analyse the data set (for different purposes), additional ethical approval (on a individual user and purpose basis) will be required. The authors would be happy to support additional ethical approval applications from researchers for access to this data set.

## Additional information

**How to cite this article:** Fattinger, S. *et al*. Deep sleep maintains learning efficiency of the human brain. *Nat. Commun.* **8,** 15405 doi: 10.1038/ncomms15405 (2017).

**Publisher’s note:** Springer Nature remains neutral with regard to jurisdictional claims in published maps and institutional affiliations.

## Supplementary Material

Supplementary Information Supplementary Figures, Supplementary Tables and Supplementary Reference
