# Author Correction: Deep sleep maintains learning efficiency of the human brain

**DOI:** 10.1038/ncomms16182

**Published:** 2018-05-25

**Authors:** Sara Fattinger, Toon T. de Beukelaar, Kathy L. Ruddy, Carina Volk, Natalie C. Heyse, Joshua A. Herbst, Richard H. R. Hahnloser, Nicole Wenderoth, Reto Huber

Nature Communications 8: Article number: 15405; DOI: 10.1038/ncomms15405 (2017); Published online 05 22 2017; Updated 05 25 2018

The originally published version of this Article contained errors in Figure 4. In panels c and d, the labeling of the light and dark blue lines was inverted. These errors have now been corrected in both the PDF and HTML versions of the Article.

