# Supplementary material for: Deep sleep maintains learning efficiency of the human brain
Source: Nat Commun. 2017 May 22;8:15405. doi: 10.1038/ncomms15405 (PMC5458149; doi:10.1038/ncomms15405)
Supplement: Supplementary Information — Supplementary Figures, Supplementary Tables and Supplementary Reference [file ncomms15405-s1.pdf]

Exclusion criteria and outlier detection

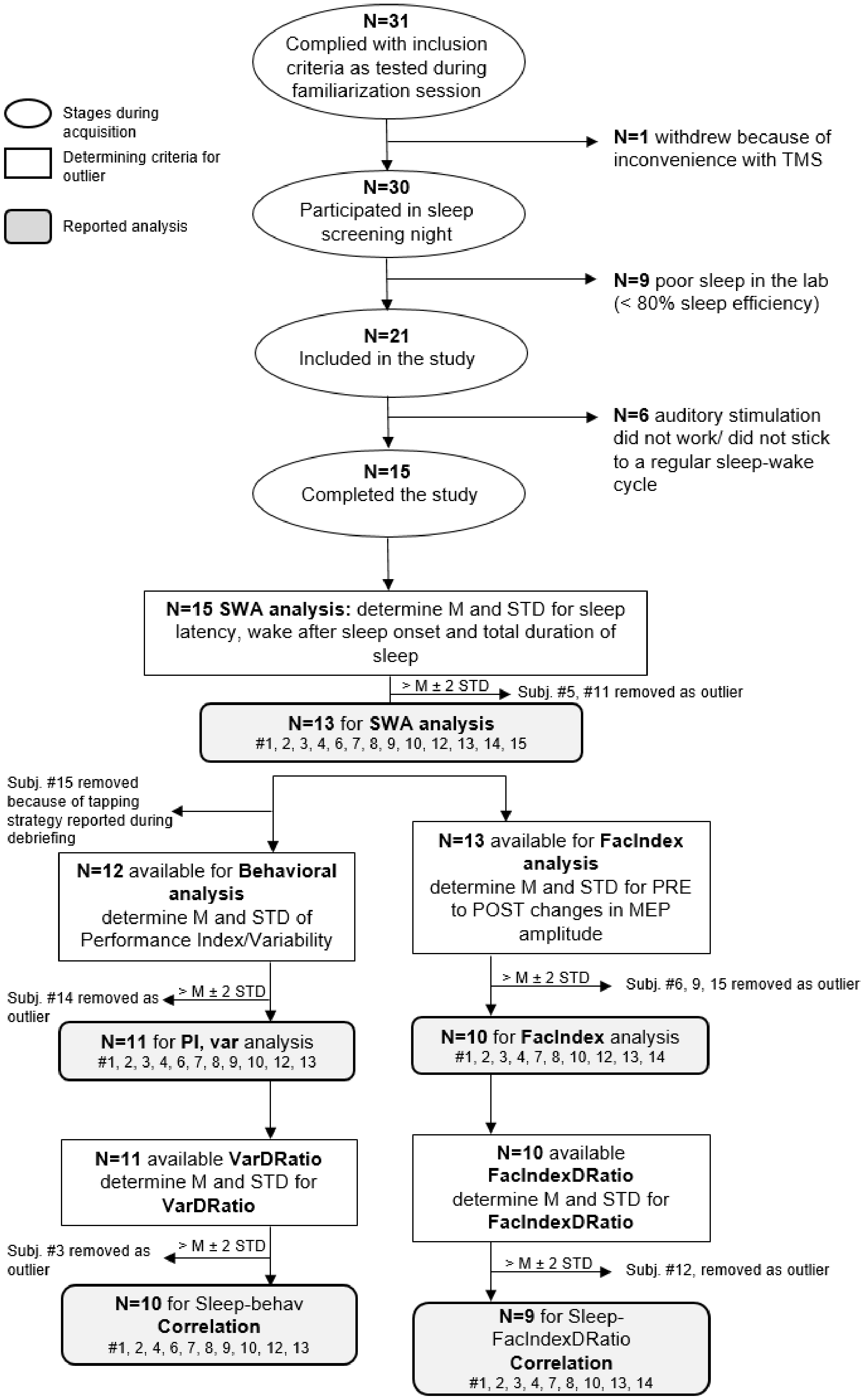

Supplementary Fig. 1

31 subjects complied with the inclusion criteria as tested during the familiarization session. The upper part of the figure (ovals) indicates how many subjects dropped-out at different stages of the experiment. The lower part indicates how the criteria for detecting outliers were defined (squares) and which participants were included in each of the analyses reported in the manuscript (shaded squares). Outliers were detected by calculating, for each parameter, mean and standard deviation (M and STD) across all participants available for the specific comparison (i.e. with potential outliers included in the dataset). An individual's data were identified as outlier when it fell outside of the mean ± 2* STD range and this individual was then removed from the statistical analysis. Outlier detection and removal was performed stepwise: We first scrutinized whether sleep quality was comparable between the two nights. This is important because the goal of our study was to disturb slow wave sleep locally while keeping the global sleep structure intact. We chose sleep latency, wake after sleep onset and total duration of sleep as key parameters and calculated the difference between the STIM and the NOSTIM night. In two subjects at least one of these three parameters exceeded the group mean ± 2*STD and these subjects were removed from further analyses because changes in the general sleep architecture might have a big influence on both behavioral and TMS measurements of motor learning. For the remaining 13 subjects, we identified outliers with regard to our TMS measurement by assessing changes in mean corticomotor excitability from PRE to POST which were then averaged across stimulation session (STIM, NOSTIM) and learning assessment (MorD1, EveD1, MorD2). We then calculated the mean and STD of these data across the 13 subjects and found that three subjects had values exceeding the mean + 2 STD leaving n = 10 for the FacIndex analysis. Next we calculated the FacIndex Δ Ratio and detected one outlier which was removed from the dataset leaving n = 9 to be included in the correlation analysis testing for associations between differences in SWA at the hotspot-electrode and the FacIndex Δ Ratio. For the behavioural data, we had to remove one subject based on the debriefing because he/she reported that he/she tapped a specific rhythm which confounds the variability measurements. After this subject was removed, our outlier detection procedure was applied: each of the behavioral parameters (Performance Index, Tapping Variability) was averaged across trials (1 to 12), stimulation session (STIM, NOSTIM) and learning assessment (MorD1, EveD1, MorD2). We then calculated the mean and STD of these data across the 12 subjects and found that one subject's variability measurement exceed the group mean by more than 2 STD. This subject was removed from both the performance Index and the Variability analysis leaving n = 11. Next we calculated the Var Δ Ratio but had to remove one participant who exceeded the outlier criterion. Thus, n = 10 subjects were included in the final correlation

analysis testing an association between SWA reductions observed at STIM versus NOSTIM and the Var Δ Ratio.

Spectral power changes between STIM and NOSTIM night

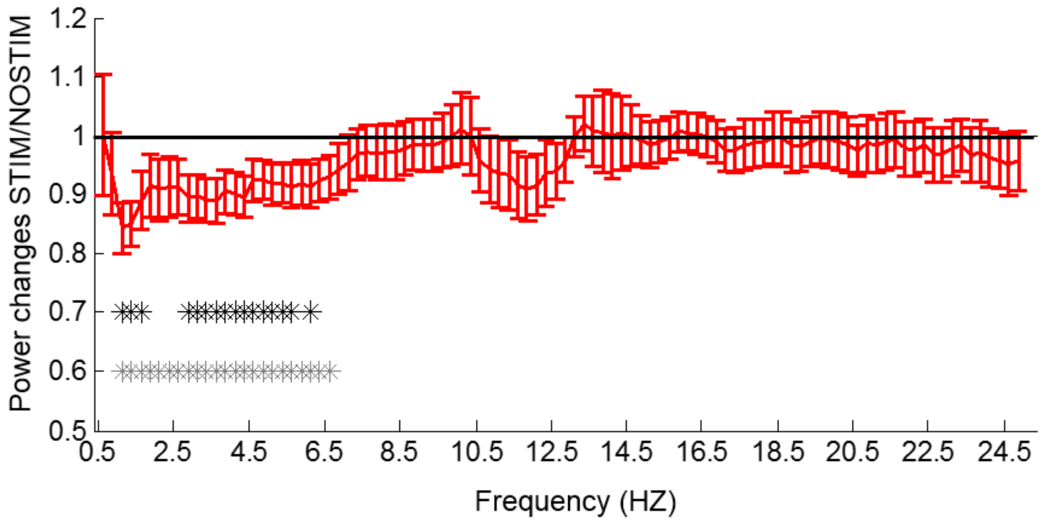

Supplementary Fig. 2

Spectral power (0.5-25Hz, divided in 0.25Hz bins) for the hotspot-electrode of the STIM night expressed relative to the NOSTIM night (mean ± SEM; 1 = spectrum power of the NOSTIM night, black stars represent frequencies with $p < 0.05$ and gray stars represent a $p < 0.1$, paired *t*-test; n=13,). Note, the strongest reduction was found for frequencies between 1-1.75Hz, however, also higher frequencies within the delta and theta range (up to 6.75Hz) showed a similar tendency.

Changes between STIM and NOSTIM night for different frequency bands

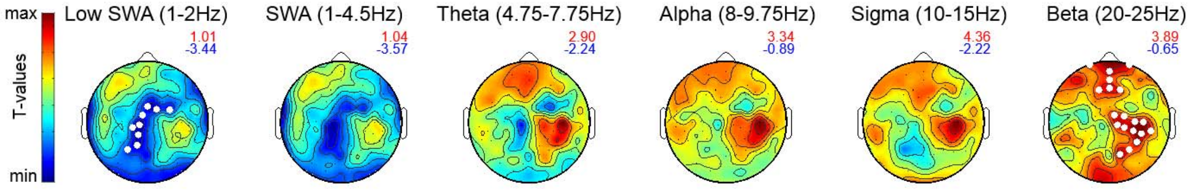

Supplementary Fig. 3

Statistical comparison (*t*-values) of power values between STIM and NOSTIM sessions for different frequency bands. Blue colors indicate a decrease and red colors an increase in power values in the STIM compared to NOSTIM session. The numbers in the upper right corner indicate the maximum (red) and minimum (blue) *t*-value for each map. Significant electrodes are marked with white dots ($p < 0.05$, paired *t*-test; n = 13, after nonparametric cluster-based statistical testing). Note, compared to low-SWA, beta power revealed an increase over frontal and right central/temporal cortices in the STIM session compared to the NOSTIM session. However, it is important to mention that power in neither of these two clusters correlated with the FacIndex Δ Ratio or Variability Δ Ratio (for a detailed overview of all correlations see Supplementary Table 7).

Changes in corticomotor excitability of all muscles

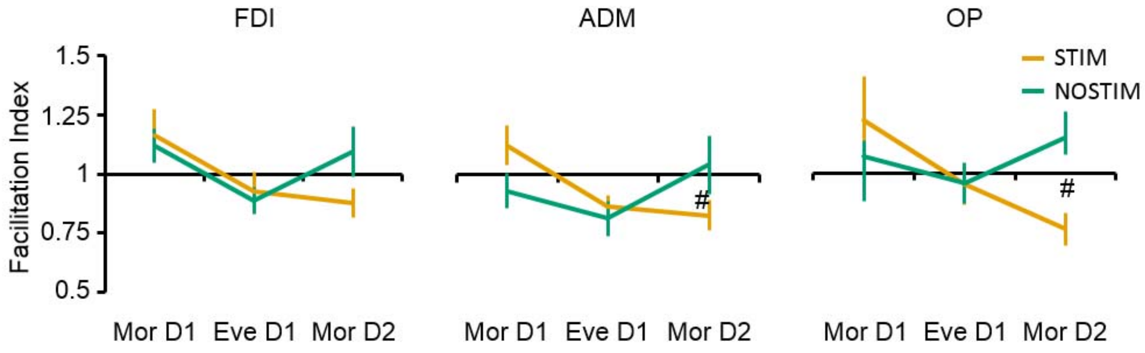

Supplementary Fig. 4

Changes in corticomotor excitability for each learning assessment were summarized by a Facilitation Index (FacIndex = $\int_{Intensity\ 1\text{-}5} MEP_{post}$ / $\int_{Intensity\ 1\text{-}5} MEP_{pre}$) and are shown for each muscle (first dorsal interosseous = FDI, abductor digiti minimi = ADM and opponens pollicis = OP). A FacIndex > 1 indicates an increase in corticomotor excitability from PRE to POST training, while a FacIndex < 1 indicates a decrease. For the ADM and OP muscles, a *stimulation session learning assessment* interaction was found (ADM: $F(2,45) = 4.56$, $p = 0.016$; OP: $F(2,45) = 4.52$, $p = 0.016$, n = 10), with pairwise comparisons indicating a significant difference between STIM and NOSTIM only on the Mor D2 timepoint (ADM: $p = 0.03$; OP: $p = 0.005$). For the FDI a similar decrease of the FacIndex from Mor D1 to Eve D1 was apparent which was only renormalized after unperturbed sleep (i.e. NOSTIM), but the interaction did not reach significance ($p = 0.09$). Vertical bars indicate SEMs. # Significant post hoc analysis STIM vs NOSTIM. All post hoc tests were corrected for multiple comparisons in line with the modified Bonferroni procedure (adjusted criterion alpha = 0.03). See methods for further details.

Facilitation Index and tapping Variability of the control experiment

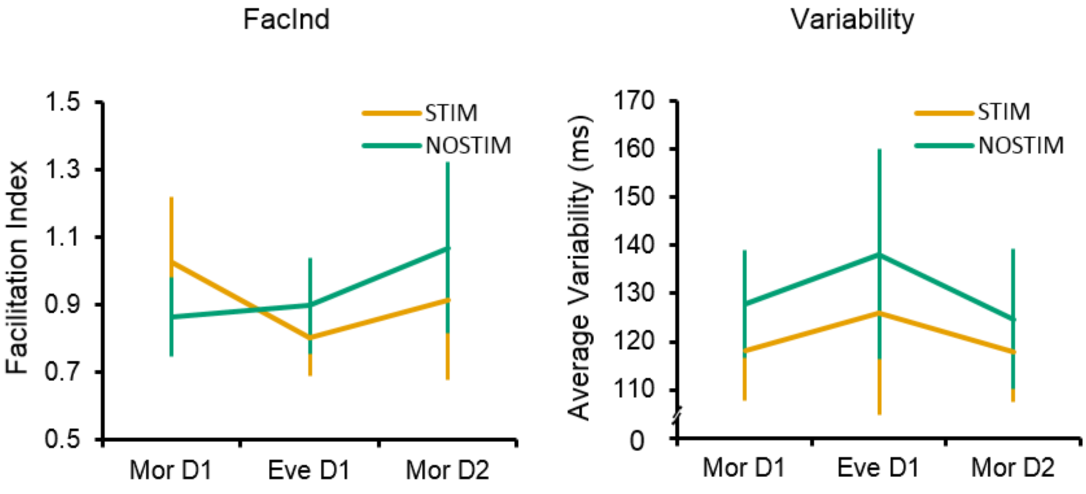

Supplementary Fig. 5

Facilitation Index and tapping Variability are shown for both the STIM (yellow) and the NOSTIM session (green, mean ± SEM, n=7). Note that the Facilitation Index was highly variable on the Morning of D1, most likely because we had limited control about the subjects' behaviour on the day before the experiment started. Importantly, the FacIndex of STIM and NOSTIM was very similar on the Evening of D1 and also overnight improvements occurred to a similar extent. Tapping Variability was slightly lower in the STIM than in the NOSTIM session, however, the pattern across the timepoints was highly consistent such that in both sessions variability was higher in the evening than in the morning measurements.

Topographical distribution of detected slow waves

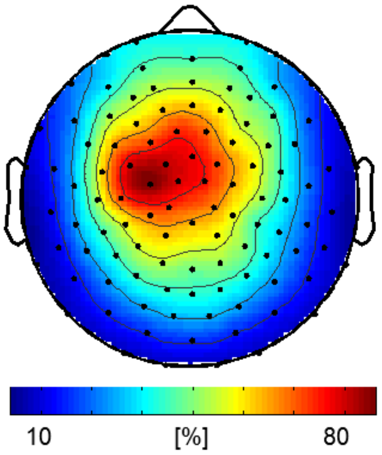

Supplementary Fig. 6

Mean percentage of slow waves (≤ -30µV) detected within a time window of ±50ms when a tone was applied for each channel (n=13). Note, the further away from the hotspot-electrode the less slow waves were detected.

Local slow wave activity changes and phase timing of tone onset

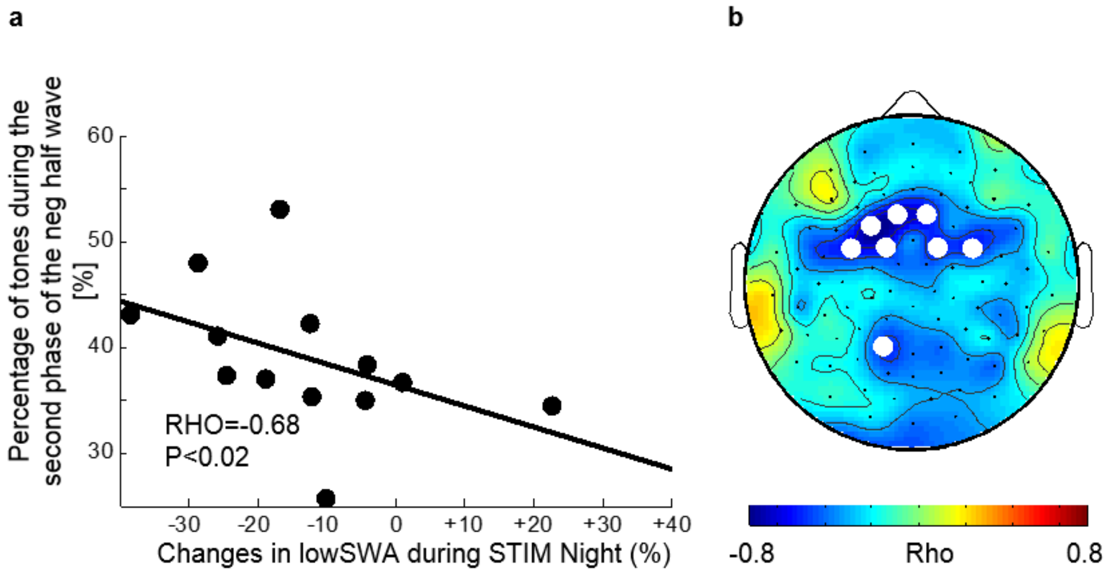

Supplementary Fig. 7

Relationship between phase timing of tone onset at the hotspot-electrode and the local reduction in lowSWA in the STIM compared to the NOSTIM night. The phase timing of the online slow wave detection algorithm was quantified offline using Hilbert transformation (based on the signal of the hotspot-electrode). On average 82% of all tones were applied during the negative phase of the slow wave cycle. We also performed a more detailed analysis of the phase timing by dividing the negative half wave into the first half of the half-wave (i.e. from the first zero crossing to the minimum) and the second half of the negative half wave (i.e. from the minimum to the subsequent zero crossing). To further determine whether phase timing of the tone might contribute to this observed variability in lowSWA changes, the percentage of tones applied during the first versus the second half wave was correlated with the lowSWA changes in the hotspot-electrode (Spearman's *rho* coefficient, n=13) as shown in (a). We found that the more tones were played during the second half of the negative half-wave, the more lowSWA was reduced in the hotspot-electrode. (b) Correlation between changes in lowSWA for all channels and the percentage of tones allocated during second half of the negative half-wave (Spearman's *rho* coefficient, white dots $p < 0.05$, uncorrected, n=13). Note: The correlation was strongest around the hotspot-electrode suggesting that the effect of the auditory stimulation was local.

Subjective sleep quality

	NOSTIM (mean±SEM)	STIM (mean±SEM)
Tiredness after sleep	4.51 ± 0.48	4.22 ± 0.37
Restfulness of sleep	4.53 ± 0.28	4.09 ± 0.23
Sleep depth *(compared to normal sleep)*	4.82 ± 0.31	4.50 ± 0.31
Sleep depth *(compared between nights)*	4.78 ± 0.30	4.22 ± 0.30

Supplementary Table 1

Summary of the subjective quality of sleep based on questionnaires. Tiredness after sleep; Restfulness of sleep: 0 = very tired, not restful; 9 = not tired, very restful. Sleep depth (compared to normal sleep): 0 = lighter sleep than normally; 9 = deeper sleep than normally. Sleep depth (compared between nights): 0 = light sleep; 9 = deep sleep. Note, repeated measures ANOVAs showed no significant difference between STIM and NOSTIM night ($p > 0.12$, n=13).

Psychological questionnaire

		NOSTIM (mean ± SEM)	STIM (mean ± SEM)
**Tiredness**	Morning 1	4.43 ± 0.32	4.69 ± 0.71
	Evening 1	4.62 ± 0.48	5.12 ± 0.42
	Morning 2	5.68 ± 0.31	5.52 ± 0.47
**Mood**	Morning 1	6.54 ± 0.61	6.93 ± 0.73
	Evening 1	6.16 ± 0.47	6.26 ± 0.37
	Morning 2	5.87 ± 0.39	6.08 ± 0.31
**Energy**	Morning 1	4.38 ± 0.40	4.12 ± 0.82
	Evening 1	4.57 ± 0.46	5.51 ± 0.23
	Morning 2	5.15 ± 0.36	5.37 ± 0.30
**Calm**	Morning 1	7.17 ± 0.80	7.48 ± 0.66
	Evening 1	7.04 ± 0.33	6.90 ± 0.35
	Morning 2	6.73 ± 0.37	6.92 ± 0.32
**Focus**	Morning 1	5.41 ± 0.41	5.98 ± 0.60
	Evening 1	6.23 ± 0.41	5.62 ± 0.31
	Morning 2	5.45 ± 0.29	5.37 ± 0.33
**Motivation**	Morning 1	3.54 ± 0.31	3.63 ± 0.46
	Evening 1	3.26 ± 0.38	3.96 ± 0.45
	Morning 2	3.75 ± 0.37	4.22 ± 0.39

Supplementary Table 2

Summary of the psychological questionnaires. Tiredness, Mood, Energy, Calm, Focus, Motivation: 0 = not tired, very bad mood, full of energy, very restless, not focused, very motivated; 10 = very tired, very good mood, no energy, very calm, very focused, not motivated. Note, mixed effect models revealed no significant difference between STIM and NOSTIM night ($p > 0.12$, n=13).

Objective measures of vigilance

	Reaction time (ms)	
	Evening	Morning
STIM	254.24 ± 31.05	249.22 ± 21.83
NOSTIM	268.52 ± 41.74	257.82 ± 52.00

Supplementary Table 3

Reaction Time of the auditory attention task which was performed in the evening and morning to assess vigilance states. Note, no significant difference between STIM and NOSTIM night was found ($p > 0.12$; paired *t*-test, n=12, for one subject the attention task was skipped in the morning, due to time restrictions (i.e. lecture attendance)).

To control for general changes in psychological conditions subjects filled in a psychological questionnaire (assessing attention, mood, concentration and motivation on a visual analog scale) prior to each learning session (Moring 1, Evening 1 and Morning 2). In addition subjects were asked to rate their subjective sleep quality (based on an analog visual scale) in the morning after the EEG recordings. There were not changes in any assessed variable between the two sessions (see Supplementary Table 1 and 2), indicating that our behavioral and neural measurements were unlikely confounded by lack of attention, motivation or other side effects typically associated with general sleep deprivation.

Moreover, subjects completed an attention task (based on an auditory oddball paradigm) in the evening before and in the morning after sleep recording (~15min before bed time and 15min after wake up) to control for vigilance state (i.e. sleep inertia). Reaction times to the deviant tones, number of missed clicks and number of wrong clicks were recorded as performance scores. Independent of the condition (NOSTIM or STIM) subjects performed equally well in all performance scores at all time points (evening and morning; all, $p > 0.12$).

Sleep architecture of the main experiment

	NONSTIM	STIM	*p*-values
**Nr of Stimulations**	0.00 ± 0.00	3143.69±354.73	
**NREMS [min]**	318.8±5.7	320.4±5.0	0.68
**N1 [min]**	21.0±2.2	24.2±1.8	0.22
**N2 [min]**	167.3±9.9	178.4±9.8	0.10
**N3 [min]**	130.5±10.6	117.9±9.8	0.05
**REMS [min]**	99.6±3.6	92.9±3.7	0.12
**TST [min]**	418.4±5.6	413.3±5.6	0.28
**SEF [%]**	93.9±0.9	93.9±0.7	0.96
**SL [min]**	12.2±2.4	12.2±2.6	0.96
**WASO [min]**	18.6±3.6	17.8±1.9	0.77
**Nr WASO**	34.1±3.6	35.4±3.0	0.72
**Nr Arousal**	24.3±2.4	23.5±2.8	0.73

Supplementary Table 4
Visually scored sleep variables for both sessions (mean ± SEM; paired *t*-test; n = 13; NREMS = NREM sleep; N1-N3 = NREMS stage 1-3; REMS = REM sleep; TST = total sleep time; SEF = sleep efficiency; SL = sleep latency; WASO = wake after sleep onset; Nr WASO = number of awakenings after sleep onset, Nr Arousal = number of arousals).

The difference between the two nights in N3, and N2 sleep did not correlate with the observed changes in the Facilitation index and variability during the finger tapping task (Spearman correlation $p > 0.35$). Moreover, the number of applied auditory stimuli during the stimulation night did not correlate with the different amounts of N3 sleep between STIM and NOSTIM nights (Spearman's rho = 0.28, p = 0.35). These results indicate that the observed difference in N3 sleep in the current study sample may not be due to the acoustic stimulation per se. However, future studies with increased stimulation effectiveness and other target areas may systematically affect N3 sleep.

Sleep architecture of the control experiment

	NONSTIM	STIM	*p*-values
**Nr of Stimulations**	00 ± 00	2663 ± 541	
**NREMS [min]**	311.76 ± 4.6	321.38 ± 6.9	0.08
**N1 [min]**	24.95 ± 2.62	21.62 ± 5.0	0.46
**N2 [min]**	191.71 ± 5.82	209.62 ± 5.0	0.02
**N3 [min]**	95.10 ± 8.25	90.38 ± 10.48	0.53
**REMS [min]**	111.57 ± 5.0	109.33 ± 6.28	0.80
**TST [min]**	423.33 ± 8.55	430.71 ± 5.61	0.33
**SEF [%]**	93.24 ± 1.24	95.14 ± 0.66	0.21
**SL [min]**	14.43 ± 3.64	10.05 ± 1.88	0.13
**WASO [min]**	19.95 ± 3.46	14.48 ± 3.23	0.30
**Nr WASO**	36.14 ± 6.85	34.14 ± 7.92	0.21

Supplementary Table 5

Control experiment: Visually scored sleep variables for both sessions (mean ± SEM; paired *t*-test; n = 7; NREMS = NREM sleep; N1-N3 = NREMS stage 1-3; REMS = REM sleep; TST = total sleep time; SEF = sleep efficiency; SL = sleep latency; WASO = wake after sleep onset; Nr WASO = number of awakenings after sleep onset).

Wrist actigraphy data

	n	Activity score/min day before the experiment	Activity score/min day of the experiment
NOSTIM	13	393.16±28.15	370.06±24.41
STIM	12	386.37±23.99	354.60±33.01
p-Values		0.57	0.94

Supplementary Table 6

Summary of the activity counts /min of the day before the first experimental session and the day of the experimental sessions (mean ± SEM, paired *t*-test, n=12). Activity was recorded with a wrist actigraph (Actiwatch, subjects 1-7 and Geneactiv subjects 8-13, in one subjects the actigraph did not work prior to the STIM session). Activity counts/min were calculated for Actiwatch recorders according to the Actiwatch software (Actiwatch. Actiware - Tutorials: http://www.actigraphy.com/devices/actiware/tutorials.html), and for Geneactiv recorders according to the algorithm described by te Lindert and Van Someren[1].

Correlation between power differences and Variability/ Facilitation Index

	Variability		Facilitation Index	
**Clusters**	**rho**	**p**	**rho**	**p**
lowSWA	0.22	0.54	-0.37	0.34
Beta front	-0.18	0.63	-0.52	0.16
Beta right cent/temp	-0.41	0.25	-0.48	0.19

Supplementary Table 7

Summary of the Spearman correlation coefficients (rho and p values, n=13) between clusters (mean over all electrodes within the cluster), revealing power differences between the two nights (considering all frequency bands from Fig. S3) and changes in Facilitation Index or Variability. For lowSWA one cluster located over the left sensory-motor cortex, and for beta power two clusters (cluster1 located over frontal cortex and cluster 2 located over right central and temporal cortices, for topographical details see Fig. S3) were included.

References

1. te Lindert, B.H.. W., Van Someren, E.J.W. Sleep estimates using microelectromechanical systems (MEMS). Sleep 36, 781–9 (2013).
